# A positive faecal immunochemical test result and its association with the incidence of rheumatoid arthritis, systemic lupus erythematosus, and psoriatic arthritis: an analysis of one-million national colorectal cancer screening programme results

**DOI:** 10.1186/s12916-022-02416-y

**Published:** 2022-07-04

**Authors:** Choong-Kyun Noh, Eunyoung Lee, Bumhee Park, Sung Soo Ahn

**Affiliations:** 1 Department of Gastroenterology, Ajou University School of Medicine, Suwon, Republic of Korea; 2 Department of Medical Sciences, Biomedical Informatics, Graduate School of Ajou University, Suwon, Republic of Korea; 3 Department of Biomedical Informatics, Ajou University School of Medicine, Suwon, Republic of Korea; 4 Office of Biostatistics, Ajou Research Institute for Innovative Medicine, Ajou University Medical Center, Suwon, Republic of Korea; 5 Division of Rheumatology, Department of Internal Medicine, Yongin Severance Hospital, Yonsei University College of Medicine, Yongin, Republic of Korea

**Keywords:** Faecal immunochemical test, Rheumatoid arthritis, Systemic lupus erythematosus, Psoriatic arthritis, Systemic inflammation

## Abstract

**Background:**

Accumulating evidence now indicates that the presence of faecal haemoglobin, in the absence of gastrointestinal bleeding, may be an indicator of systemic inflammation and is linked to the development of human diseases. We evaluated whether a positive faecal immunochemical test (FIT) is associated with the development of immune-mediated inflammatory diseases (IMIDs).

**Methods:**

Data from the nationwide colorectal cancer screening programme from 2009 to 2013 were used. Participants (*n*=8,646,887) were divided into FIT (+) and FIT (-) groups by performing a 1:1 random sampling matched by age and sex. Participants with concurrent haemorrhoids, colorectal cancer (CRC), inflammatory bowel disease (IBD), and missed CRC and IBD were excluded using the colonoscopy results, ICD-10 codes, and the special exemption code (V code). Endpoints were the incidence of IMIDs (rheumatoid arthritis [RA], systemic lupus erythematosus [SLE], and psoriatic arthritis [PsA]) after FIT.

**Results:**

Of the 1,044,955 eligible participants, 229,594 and 815,361 individuals were included in the FIT (+) and the FIT (−) groups, respectively. During the mean follow-up period of 7.59 years, a total of 7645 (incidence rate [IR] 9.56/10,000 person-years [PY]), 208 (IR 0.26/10,000 PY), and 101 (IR 0.13/10,000 PY) patients were diagnosed with RA, SLE, and PsA, respectively. An adjusted Cox analysis demonstrated that FIT positivity conferred a 1.16 (95% confidence interval [CI] 1.09–1.24, *p*<0.001) times greater risk of developing RA. Kaplan–Meier analysis in the 1:2 propensity-score matched population also confirmed these results (hazard ratio [HR] 1.18, 95% CI 1.10–1.27, *p*<0.001).

**Conclusions:**

Positive FIT is associated with increased risk of RA in the general population, corroborating that aberrancies of gut mucosa are associated with the development of IMIDs. Vigilant monitoring and early referral to a specialist upon medical suspicion is required in this population.

**Trial registration:**

Retrospectively registered.

**Graphical abstract:**

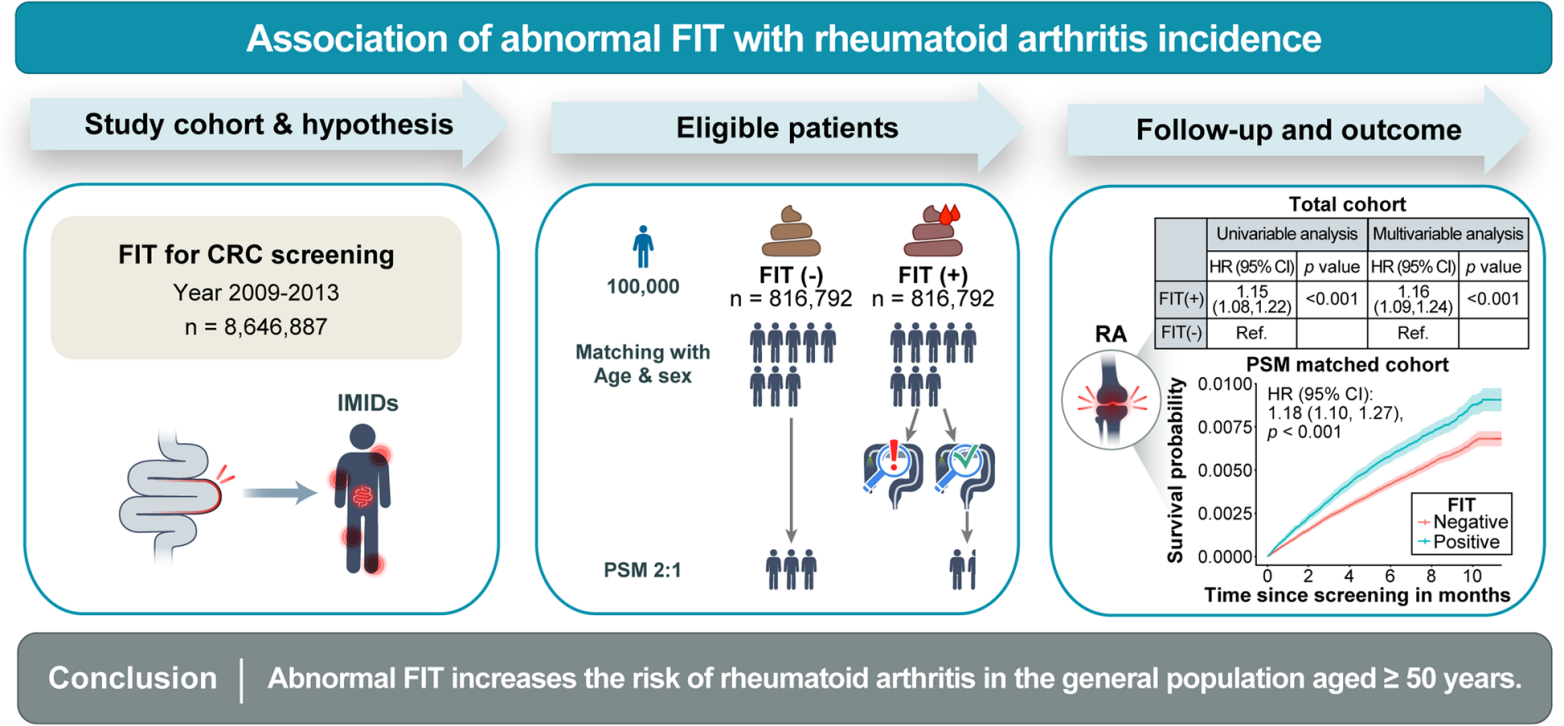

## Background

The human intestine primarily functions as a tract for the intake of food and nutrients and to eliminate waste produced from the human body. Nonetheless, it is increasingly recognised that the human intestine also serves as an active organ, playing a pivotal role in the development of systemic autoimmunity [1]. The literature suggests that a complex relationship exists between the gut and the immune system; importantly, disruption of local bowel homeostasis and changes in the gut microbiota are now thought to be a crucial link between the gut and altered immunity [2, 3]. The breach of localised gut immunity in the physiological state causes changes in the balance of pro- and anti-inflammatory mediators in the gut microenvironment and leads to extra-intestinal abnormal immune cell trafficking [4]. In addition, the expansion of pathogenic microbiota has been shown to play a detrimental role in normal immunity, by promoting an inflammatory milieu [5]. Consistently, there is a growing body of evidence indicating that injury to the bowel barrier function is associated with the development of human diseases.

Immune-mediated inflammatory diseases (IMIDs) are a heterogeneous group of disorders, such as rheumatoid arthritis (RA), ankylosing spondylitis, psoriatic arthritis (PsA), and systemic lupus erythematosus (SLE) that can affect virtually every organ [6]. Genetic and environmental factors contribute to the evolution of IMIDs, and their diagnoses are discriminated according to the tissues affected and their characteristic clinical and laboratory features. Although IMIDs are traditionally considered uncommon, their incidence is reported to be increasing continuously with a significant impact on patient morbidity and mortality globally [7]. Notably, even though differences in the key pathogenesis of these diseases are present, a common immunological feature of IMIDs is heightened inflammatory and blunted anti-inflammatory responses. For example, inflammatory cytokines are overexpressed, and anti-inflammatory cytokines are downregulated in IMIDs and a disease-specific polarisation of CD4^+^ T cells, a critical regulator of adaptive immunity, is also observed [8]. Thus, current therapeutic approaches in IMIDs mainly target activated immunity and inflammatory cytokines.

Recent studies have revealed a relationship between the presence of haemoglobin in the faeces and diseases associated with systemic inflammation [9]. Raised faecal haemoglobin concentrations without clear evidence of gastrointestinal bleeding, which can be measured by the faecal immunochemical test (FIT), reflect subclinical inflammation. This suggests the potential future applications of FIT as an early detection tool for chronic diseases, in the absence of obvious source of gastrointestinal bleeding. In addition, since the intestinal tract has an essential role in the onset of aberrant immune responses, it could be hypothesised that the incidence of IMIDs is increased in those with gut mucosal abnormality. Nevertheless, to the best of our knowledge, differences in the occurrence of IMIDs according to FIT test result have not been well studied. Therefore, the objective of this study was (i) to evaluate whether positive FIT is associated with increased risk of IMIDs and (ii) to evaluate predictive factors of RA occurrence in the general population that participated in a nationwide colorectal cancer screening programme.

## Methods

### Study cohort and database

This study was conducted using the Korean National Cancer Screening Program (KNCSP) and the National Health Insurance Sharing Service-National Health Information Database (NHIS-NHID). The Korean NHIS covers approximately 97% of the total population and provides medical services [10]. A detailed description of the KNCSP system is available [11]. This database is based on the government-managed national health insurance system, and the entire population is included, according to the criteria for each cancer [12]. For colorectal cancer (CRC) screening in the KNCSP, the FIT, which is more sensitive than a guaiac-based faecal occult blood test for CRC [13–16], is used for individuals aged 50 years or older, annually. When the FIT is positive, the Korean National Health Care System covers the subsequent examination, either colonoscopy or double-contrast barium enema, chosen by the individual [17]. The total cohort was selected for the most recent data, while keeping the trace as long as possible. Therefore, we finally obtained the 2009–2013 dataset for the population, and we followed the selected participants up to December 21, 2019. The study protocol was approved by the Ajou University hospital’s institutional review board (approval no. AJIRB-MED-EXP-20-479), which waived the requirement for individual informed consent owing to the use of a de-identified dataset.

### Enrolled participants and eligibility criteria

**Fig. 1:**
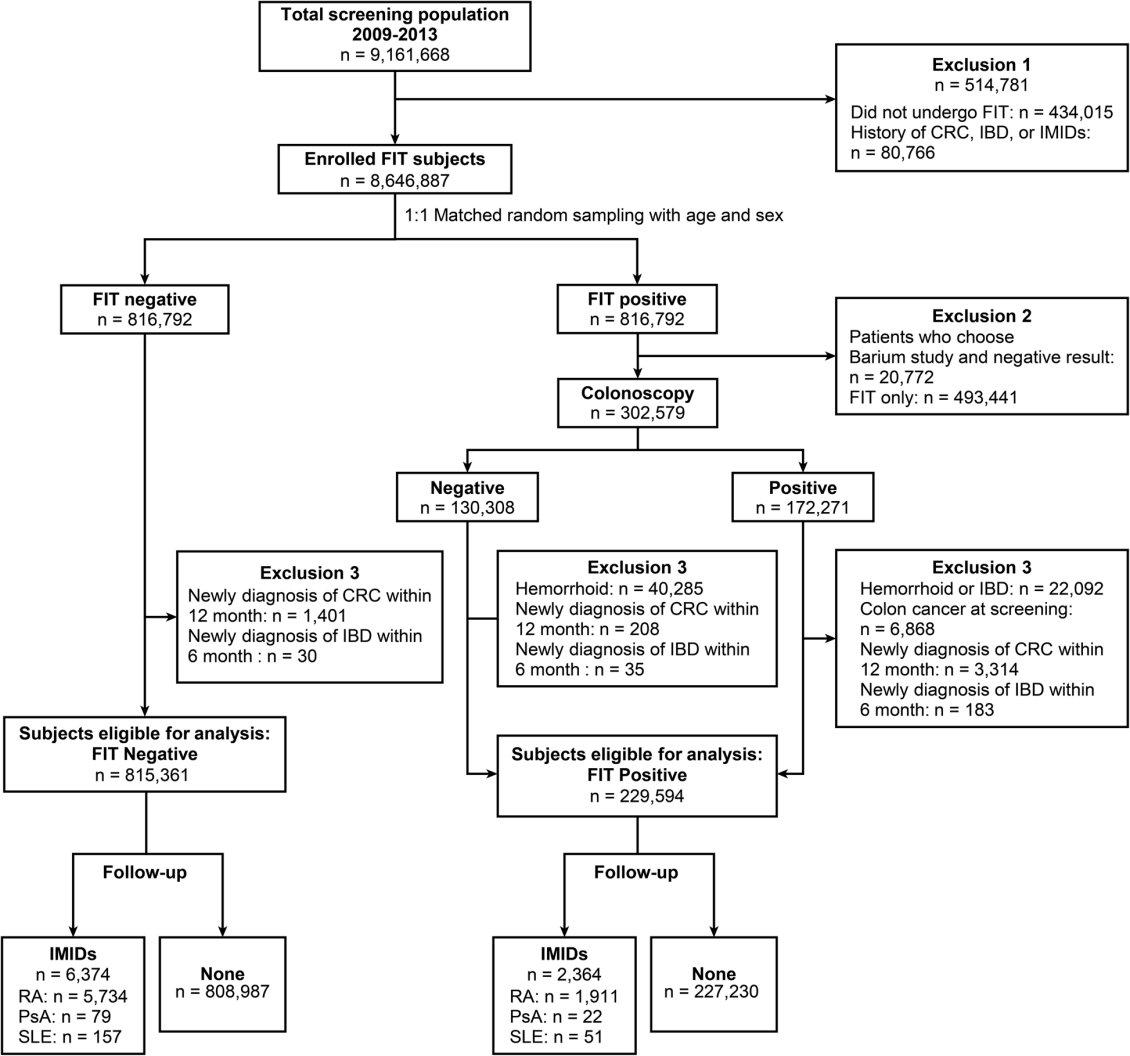
Flowchart showing participant selection. *FIT* faecal immunochemical test, *CRC* colorectal cancer, *IMID* immune-mediated inflammatory diseases, *RA* rheumatoid arthritis, *PsA* psoriatic arthritis, *SLE* systemic lupus erythematosus, *IBD* inflammatory bowel disease

Of the screened participants, we first excluded those that did not undergo FIT, and those who had a history of CRC, inflammatory bowel disease (IBD), and IMIDs. After identifying 816,792 patients with positive FIT results (the FIT positive group, FIT [+]), a 1:1 random sampling matched by age and sex was done for the FIT negative group (FIT [−]). The FIT (+) group was analysed after excluding those (1) who did not undergo colonoscopy; (2) who had a diagnosis of haemorrhoids, IBD, or CRC based on the result of colonoscopy; (3) newly diagnosed with IBD or CRC after FIT, within 6 and 12 months, respectively, according to the International Statistical Classification of Diseases, Tenth revision codes (ICD-10 codes) to exclude the possibility of missed IBD and CRC [18]. Likewise, participants who were diagnosed with IBD within 6 months and CRC within 12 months of the FIT were excluded from the FIT (−) group (Fig. 1).

### Identification of CRC, IBD, and IMIDs

The NHIS-NHID is the health claim database submitted by medical institutions for their medical services, including inpatient and outpatient visits. It contains diagnosis codes, treatments, prescriptions, and medical expenses. We used the primary and secondary diagnosis codes to identify patients with CRC, IBD, and IMIDs. Moreover, to improve diagnosis accuracy, we also found the prescription history of disease-modifying anti-rheumatic drugs for RA and special exemption codes (V Code) for IBD, SLE, and PsA. The Korean government gives special exemption codes to financially help patients with rare intractable diseases, by reducing coinsurance rates of medical expenses by up to 90%, which could be used to select patients with the corresponding disorders correctly. Since the national health insurance provides a subsidy payment for the treatment of these diseases, they are certified by the attending physician based on standard diagnostic criteria defined by the national health insurance [19].

Patients who developed CRC and IBD were determined using the ICD-10 codes of CRC (C18–C20) and IBD (K50–K51), and IBD cases that were also assigned special exemption codes (V code) of V130 (Crohn’s disease [CD]) and V131 (ulcerative colitis [UC]) were selected [20]. IMIDs were identified according to the following criteria, which were defined in previous studies: (1) RA: M05–M06 and the prescription of disease-modifying anti-rheumatic drugs (DMARDs) [21], (2) systemic lupus erythematosus: M32 and V136 [22], and (3) psoriatic arthritis: L40.5, M07.0–M07.3, M09.0, and V237 [23].

### Sample collection for FIT

All participants for CRC screening in KNCSP were instructed to collect and submit their faecal samples for FIT. Participants were guided to sample the faeces before contact with urine or water and return them to the screening centre as soon as possible. The faecal sample was recommended for collection on the day of the visit to the centre. Results of FIT testing for CRC screening via KNCSP was determined by either qualitative (negative/positive) or quantitative methods. The former is used with commercially available qualitative FIT kits (FOBtest, Humasis Co., Korea, SD Bioline FOB, SD Co., Korea, ASAN Easy Test FOB, Asan Pharm Co., Korea, and OC-Hemocatch Lignt TM, Eiken Chemical Co., Japan, cut-off value are 50 ng/ml, 30 ng/ml, 50 ng/ml, and 50 ng/ml, respectively, and the latter is office-based analysis with immunochromatographic technology (latex agglutination nephelometric immunoassay, Eiken Chemical Co. Japan). Each screening centre can select a method, and the selected method must be recorded for each subject. If a quantitative method is used, the value of measurement for faecal haemoglobin content, as well as the cut-off point of the relevant institution must be recorded. In 2009, 72.8% of participants underwent FIT via a qualitative method [24, 25]. The results were then, finally, categorised into negative and positive according to the cut-off points of each test kit.

### Definition of variables

All the participants for KNCSP completed a self-administered questionnaire which included demographic and socioeconomic information, clinical information such as lifestyle measures (smoking, alcohol drinking, and exercise), medical history, and family history. They also voluntarily completed a comprehensive medical examination that included weight, height, blood pressure, blood tests, and urinalysis. Variables obtained from the KNCSP results were sex, age, body mass index (BMI), smoking status, alcohol drinking status, exercise, insurance type (national health insurance or medical aid), income status (divided into 1st, 2nd, 3rd, 4th, and 5th quantiles: the 1st quantile indicates those with the lowest income), comorbidities based on laboratory findings and concurrent medication (hypertension, diabetes, and dyslipidaemia), and laboratory measures (blood haemoglobin, serum creatinine, serum total cholesterol, serum triglycerides, serum high-density lipoprotein [HDL]-cholesterol, and serum low-density lipoprotein [LDL]-cholesterol). Additionally, we categorised the laboratory measures into groups within and beyond the medical decision-making limit, based on the National Health check-up cut-off values (Table 1) [26].

**Table 1:** The medical decision-making limit for laboratory measures

Laboratory measures	Limit values	
	Male	Female
Blood haemoglobin, g/dL	≥13	≥12
Serum creatinine, mg/dL	≤1.5	
Serum total cholesterol, mg/dL	<200	
Serum triglyceride, mg/dL	<150	
Serum HDL-cholesterol, mg/dL	≥60	
Serum LDL-cholesterol, mg/dL	<130	

The medical decision-making limit of laboratory measures was defined according to the values proposed by the National Health check-up programme

*HDL* high-density lipoprotein, *LDL* low-density lipoprotein

Participants invited to participate in KNSCP were tested at approximately 4,300 centres, designated by the government (general hospitals, hospitals, and clinics). For quality control, the Ministry of Health and Welfare implemented the quality management project of the KNCSP, since 2008. Moreover, accreditation of the centres is evaluated annually through the Korean Laboratory Accreditation Program, attested by the Korean Society of Laboratory Medicine [27, 28].

### Statistical analysis

Baseline characteristics are presented as median (interquartile range). The differences between the FIT (+) and FIT (−) groups were compared using the Mann-Whitney U test for continuous variables and the Chi-squared test for categorical variables. The incidence rates (IRs) per 10,000 person-years (PY) were estimated with 95% confidence intervals (CIs) by taking the total number of new cases and dividing that by the sum of person-time of the at-risk population. Age and sex-adjusted IR was also calculated to remove the effect of the age and sex differences in the groups. Consequently, for further analysis, the incidences of RA were stratified by three different time cut-offs (<1 year, 1–5 years, ≥5 years), and crude IRs and adjusted IRs were also estimated. Univariate and multivariable Cox proportional hazard regression models were used to examine risk factors associated with the incidence of RA, and the proportionality assumption was tested. Follow-up duration was defined from the FIT date to the first date when the diagnoses of IMIDs were established or to the last follow-up date. Only complete case data without missing values were included in the Cox analysis. Additionally, for sensitivity analysis, we performed propensity-score matching (PSM) at a ratio of 1:2 nearest-neighbour matching algorithm to adjust for all covariates described above. With this matched validation sample, Kaplan-Meier curves of FIT (+) and FIT (−) were plotted and the difference in incidence was tested with the log-rank test. All statistical analyses were two-sided and performed using SAS statistical software, version 9.4 (SAS Institute, Cary, NC, USA), and *p*<0.05 was considered statistically significant.

## Results

### Baseline characteristics of the FIT (+) and FIT (−) groups

Of the 1,044,955 participants included, 229,594 and 815,361 individuals were categorised into the FIT (+) and the FIT (-) groups, respectively (Table 2). Among them, 54.1% were male, the mean (standard deviation [SD]) age was 62.1 (8.6 years), and the median BMI was 24.0 kg/m^2^. Dyslipidaemia was the most common comorbidity, observed in 32.9% of the patients. Comparison of the baseline characteristics of the FIT (+) and FIT (−) groups revealed that all the investigated clinical parameters and laboratory measures differed between the groups.

**Table 2:** Baseline characteristics of the study population

	Total cohort ***n***=1,044,955	FIT (−) ***n***=815,361	FIT (+) ***n***=229,594	***p*** value
**Clinical parameter**				
Sex				<0.001
Male	565,280 (54.1)	436,092 (53.5)	129,188 (56.3)	
Female	479,675 (45.9)	379,269 (46.5)	100,406 (43.7)	
Age group at screening, years				<0.001
50–54	257,621 (24.7)	194,292 (23.8)	63,329 (27.6)	
55–59	199,710 (19.1)	152,024 (18.6)	47,686 (20.8)	
60–64	205,867 (19.7)	158,717 (19.5)	47,150 (20.5)	
65–69	146,656 (14.0)	114,319 (14.0)	32,337 (14.1)	
≥70	235,101 (22.5)	196,009 (24.1)	39,092 (17.0)	
BMI, kg/m^2^	24.0 [22.1,26.0]	24.0 [22.1,26.0]	24.1 [22.2,26.1]	<0.001
Smoking status				<0.001
No	754,426 (83.9)	592,256 (84.0)	162,170 (83.4)	
Yes	145,186 (16.1)	112,858 (16.0)	32,328 (16.6)	
Alcohol drinking				<0.001
No	709,427 (78.9)	559,665 (79.4)	149,762 (77.0)	
Yes	190,055 (21.1)	145,337 (20.6)	44,718 (23.0)	
Exercise				<0.001
No	297,913 (33.1)	236,383 (33.5)	61,530 (31.6)	
Yes	601,690 (66.9)	468,718 (66.5)	132,972 (68.4)	
Insurance type				<0.001
Medical aid	44,650 (4.3)	35,631 (4.4)	9,019 (3.9)	
NHI	100,265 (95.7)	779,696 (95.6)	220,569 (96.1)	
Income status				<0.001
1st quantile	208,136 (20.5)	162,340 (20.5)	45,796 (20.5)	
2nd quantile	145,115 (14.3)	111,690 (14.1)	33,425 (15.0)	
3rd quantile	170,324 (16.8)	131,602 (16.6)	38,722 (17.3)	
4th quantile	207,238 (20.4)	161,637 (20.4)	45,601 (20.4)	
5th quantile	285,505 (28.0)	225,514 (28.4)	59,991 (26.8)	
Comorbidities				
Hypertension				<0.001
No	656,727 (87.8)	518,754 (93.4)	137,973 (92.5)	
Yes	47,872 (12.2)	36,644 (6.6)	11,228 (7.5)	
Diabetes				<0.001
No	656,727 (93.2)	518,754 (93.4)	137,973 (92.5)	
Yes	47,872 (6.8)	36,644 (6.6)	11,228 (7.5)	
Dyslipidaemia				<0.001
No	501,128 (67.1)	397,006 (67.5)	104,122 (65.4)	
Yes	246,187 (32.9)	191,200 (32.5)	54,987 (34.6)	
**Laboratory measures**				
Blood haemoglobin, g/dL	13.8 [12.8,14.9]	13.8 [12.9,14.9]	13.8 [12.8,14.8]	<0.001
Serum creatinine, mg/dL	0.9 [0.7,1.0]	0.9 [0.7,1.0]	0.9 [0.8,1.0]	<0.001
Serum total cholesterol, mg/dL	197 [172,223]	196 [172,222]	198 [173,224]	<0.001
Serum triglycerides, mg/dL	116 [82,167]	116 [82,167]	116 [81,168]	0.011
Serum HDL-cholesterol, mg/dL	52 [44,62]	52 [44,61]	52 [44,62]	<0.001
Serum LDL-cholesterol, mg/dL	116 [93,140]	116 [93,140]	117 [94,141]	<0.001

Data presented are shown as median [interquartile range] or number (%)

*FIT* faecal immunochemical test, *BMI* body mass index, *NHI* national health insurance, *HDL* high-density lipoprotein, *LDL* low-density lipoprotein

### Incidence of IMIDs at follow-up after FIT

During the mean follow-up period of 7.6 years (SD 1.8), a total of 7645 (IR 9.56/10,000 PY), 208 (IR 0.26/10,000 PY), and 101 (IR 0.13/10,000 PY) were diagnosed with RA, SLE, and PsA, respectively. Among the IMIDs, the IR of RA and SLE were higher in the FIT (+) group compared to the FIT (−) group. When adjustment was made by age and sex, only the incidence of RA was, numerically, higher in the FIT (+) group than in the FIT (−) group, but a statistical significance was not reached (*p*=0.057) (Table 3).

**Table 3:** Incidence rates of immune-mediated inflammatory disease between the faecal immunochemical test (−) and faecal immunochemical test (+) groups

Groups	Total number (%)			IR (95% CI)			Adjusted IR (95% CI)^a^		
	RA	SLE	PsA	RA	SLE	PsA	RA	SLE	PsA
Overall	7645 (0.73)	208 (0.02)	101 (0.01)	9.56 (9.35, 9.78)	0.26 (0.23, 0.30)	0.13 (0.10, 0.15)	-	-	-
FIT (−) group (Ref.)	5734 (0.70)	157 (0.02)	79 (0.01)	9.28 (9.04, 9.52)	0.25 (0.22, 0.30)	0.13 (0.10, 0.16)	8.96 (8.21, 9.78)	0.23 (0.19, 0.28)	0.14 (0.06, 0.34)
FIT (+) group	1911 (0.83)	51 (0.02)	22 (0.01)	10.52 (10.06, 11.00)	0.28 (0.21, 0.37)	0.12 (0.08, 0.18)	10.11 (9.26, 11.03)	0.20 (0.17, 0.25)	0.09 (0.04, 0.21)
*p *value				0.929	0.944	0.969	0.057	0.374	0.493

*FIT* faecal immunochemical test, *IR* incidence rate per 10,000 person-years, *CI* confidence interval, *RA* rheumatoid arthritis, *SLE* systemic lupus erythematosus, *PsA* psoriatic arthritis

^a^Age and sex adjusted

In those who developed RA, the IR of RA was highest in the first year (IR 13.06/10,000 PY, 95% CI 12.38, 13.77), and decreased over time irrespective of seropositivity. A greater IR of RA in the first year was observed consistently in the FIT (+) group compared to the FIT (−) group, but it was not statistically significant (Table 4).

**Table 4:** Incidence rates of rheumatoid arthritis according to different time intervals and seropositivity

Groups	Total number			IR (95% CI)			Adjusted IR (95% CI)^a^		
	<1 year	1–5 years	≥5 years	<1 year	1–5 years	≥5 years	<1 year	1–5 years	≥5 years
**Overall (*n*=7645)**									
Overall	1359	4173	2113	13.06 (12.38, 13.77)	8.21 (7.97, 8.46)	2.70 (2.59, 2.82)	-	-	-
FIT (-) group (Ref.)	1038	3113	1583	12.78 (12.03, 13.59)	7.87 (7.59, 8.15)	2.62 (2.50, 2.75)	11.89 (10.56, 13.38)	7.56 (6.85, 8.33)	2.60 (2.37, 2.86)
FIT (+) Group	321	1060	530	14.02 (12.57, 15.64)	9.43 (8.88, 10.01)	2.97 (2.72, 3.23)	13.27 (11.79, 14.93)	8.88 (8.06, 9.80)	2.92 (2.65, 3.21)
*p* value				0.948	0.898	0.931	0.196	0.022	0.094
**Seropositive RA (*n*=3184)**									
Overall	553	1715	916	5.31 (4.89, 5.77)	3.37 (3.21, 3.53)	1.17 (1.09, 1.24)	-	-	-
FIT (−) group (Ref.)	428	1287	698	5.27 (4.80, 5.80)	3.25 (3.07, 3.43)	1.15 (1.07, 1.24)	4.94 (3.77, 6.47)	3.16 (2.84, 3.52)	1.15 (1.06, 1.25)
FIT (+) Group	125	428	218	5.46 (4.58, 6.51)	3.80 (3.45, 4.18)	1.21 (1.06, 1.39)	5.44 (4.15, 7.13)	3.64 (3.27, 4.06)	1.20 (1.10, 1.31)
*p* value				0.980	0.912	0.970	0.622	0.069	0.457
**Seronegative RA (*n*=4461)**									
Overall	806	2458	1197	7.74 (7.23, 8.30)	4.83 (4.64, 5.03)	1.53 (1.44, 1.61)	-	-	-
FIT (−) group (Ref.)	610	1826	885	7.51 (6.94, 8.13)	4.61 (4.40, 4.82)	1.46 (1.37, 1.56)	7.04 (6.01, 8.26)	4.39 (3.91, 4.93)	1.45 (1.27, 1.65)
FIT (+) Group	196	632	312	8.56 (7.44, 9.85)	5.61 (5.19, 6.07)	1.74 (1.56, 1.95)	7.51 (6.40, 8.80)	5.19 (4.62, 5.83)	1.70 (1.49, 1.93)
*p *value				0.927	0.889	0.902	0.581	0.046	0.087

*RA* rheumatoid arthritis, *IR* incidence rate per 10,000 person-years, *CI* confidence interval, *FIT* faecal immunochemical test

^a^Age and sex adjusted

Analysis of the incidence of RA according to age and sex showed a gradual increase until the age of 65–69 and decline in those ≥70 in both sexes. The IR of RA was more than two times higher in women compared to men in all age groups (Table 5).

**Table 5:** Incidence rate and 95% confidence intervals of immune-mediated inflammatory diseases stratified by sex and age

	RA	SLE	PsA
**Male, years**			
Age 50–54	6.05 (4.86, 7.53)	0.13 (0.11, 0.15)	0.16 (0.12, 0.21)
Age 55–59	6.27 (5.06, 7.77)	0.12 (0.1, 0.14)	0.15 (0.12, 0.2)
Age 60–64	6.98 (5.66, 8.61)	0.12 (0.1, 0.14)	0.13 (0.1, 0.18)
Age 65–69	7.13 (5.79, 8.78)	0.13 (0.11, 0.15)	0.09 (0.07, 0.12)
Age ≥70	5.72 (4.66, 7.03)	0.11 (0.09, 0.13)	0.14 (0.1, 0.18)
**Female, years**			
Age 50–54	12.52 (10.21, 15.36)	0.44 (0.38, 0.51)	0.13 (0.1, 0.17)
Age 55–59	12.98 (10.55, 15.97)	0.4 (0.34, 0.46)	0.12 (0.09, 0.16)
Age 60–64	14.45 (11.69, 17.87)	0.41 (0.35, 0.48)	0.11 (0.08, 0.14)
Age 65–69	14.76 (11.91, 18.29)	0.44 (0.38, 0.51)	0.07 (0.05, 0.09)
Age ≥70	11.84 (9.52, 14.73)	0.37 (0.32, 0.43)	0.11 (0.08, 0.14)

Values indicate IR and 95% CI in parenthesis

*RA* rheumatoid arthritis, *SLE* systemic lupus erythematosus, *PsA* psoriatic arthritis, *IR* incidence rate per 10,000 person-years, *CI* confidence interval

### Clinical and laboratory factors associated with RA development

Adjusted Cox analysis demonstrated that FIT positivity (hazard ratio [HR] 1.16, 95% CI 1.09, 1.24, *p*<0.001), female sex (HR 2.15, 95% CI 2.01, 2.29, *p*<0.001), all age groups under 70 years, and serum HDL-cholesterol < 60 mg/dL (HR 1.10, 95% CI 1.01, 1.21, *p*=0.031) were associated with increased risk of developing RA, whereas alcohol drinking (HR 0.87, 95% CI 0.80, 0.95, *p*=0.001) was inversely associated with the risk of RA (Table 6).

**Table 6:** Clinical parameters and laboratory measures associated with the occurrence of rheumatoid arthritis

	Univariable analysis		Multivariable analysis	
	HR (95% CI)	***p*** value	HR (95% CI)	***p*** value
FIT result				
Positive	1.15 (1.08, 1.22)	<0.001	1.16 (1.09, 1.24)	<0.001
Negative	Ref.		Ref.	
Sex				
Male	Ref.		Ref.	
Female	2.19 (2.07, 2.31)	<0.001	2.15 (2.01, 2.29)	<0.001
Age group at screening, years				
50–54	1.24 (1.14, 1.35)	<0.001	1.23 (1.13, 1.34)	<0.001
55–59	1.19 (1.08, 1.30)	<0.001	1.22 (1.11, 1.34)	<0.001
60–64	1.26 (1.15, 1.37)	<0.001	1.28 (1.18, 1.40)	<0.001
65–69	1.25 (1.13, 1.38)	<0.001	1.29 (1.17, 1.42)	<0.001
≥70	Ref.		Ref.	
BMI	1.00 (0.99, 1.01)	0.664	1.00 (0.99, 1.01)	0.750
Smoking status				
No	Ref.		Ref.	
Yes	0.67 (0.62, 0.73)	<0.001	1.04 (0.94, 1.14)	0.460
Alcohol drinking				
No	Ref.		Ref.	
Yes	0.61 (0.57, 0.66)	<0.001	0.87 (0.80, 0.95)	0.001
Exercise				
No	1.05 (1, 10.12)	0.068	1.02 (0.97, 1.08)	0.444
Yes	Ref.		Ref.	
Insurance type				
Medial aid	Ref.		Ref.	
NHI	0.98 (0.69, 1.38)	0.890	1.06 (0.75, 1.49)	0.744
Hypertension				
No	Ref.		Ref.	
Yes	0.92 (0.84, 1.01)	0.070	0.98 (0.9, 1.08)	0.683
Diabetes				
No	Ref.		Ref.	
Yes	0.79 (0.69, 0.89)	<0.001	0.89 (0.78, 1.01)	0.076
Dyslipidaemia				
No	Ref.		Ref.	
Yes	0.95 (0.89, 1.00)	0.068	1.00 (0.91, 1.10)	0.975
Blood haemoglobin <13 g/dL for male and <12 g/dL for female				
No	Ref.		Ref.	
Yes	1.62 (1.19, 2.21)	0.002	1.14 (0.84, 1.55)	0.406
Serum creatinine > 1.5 mg/dL				
No	Ref.		Ref.	
Yes	0.92 (0.73, 1.16)	0.480	1.08 (0.86, 1.36)	0.515
Serum total cholesterol ≥ 200 mg/dL				
No	Ref.		Ref.	
Yes	1.03 (0.96, 1.10)	0.404	0.96 (0.86, 1.06)	0.386
Serum triglyceride ≥ 150 mg/dL				
No	Ref.		Ref.	
Yes	0.85 (0.79, 0.92)	<0.001	0.93 (0.84, 1.02)	0.129
Serum HDL-cholesterol < 60 mg/dL				
No	Ref.		Ref.	
Yes	0.95 (0.88, 1.03)	0.248	1.10 (1.01, 1.21)	0.031
Serum LDL-cholesterol ≥130 mg/dL				
No	Ref.		Ref.	
Yes	1.04 (0.97, 1.11)	0.276	0.95 (0.86, 1.06)	0.384

*HR* hazard ratio, *CI* confidence interval, *FIT* faecal immunochemical test, *BMI* body mass index, *NHI* national health insurance, *HDL* high-density lipoprotein, *LDL* low-density lipoprotein

### Comparison of the incidence of RA in the matched population

**Fig. 2:**
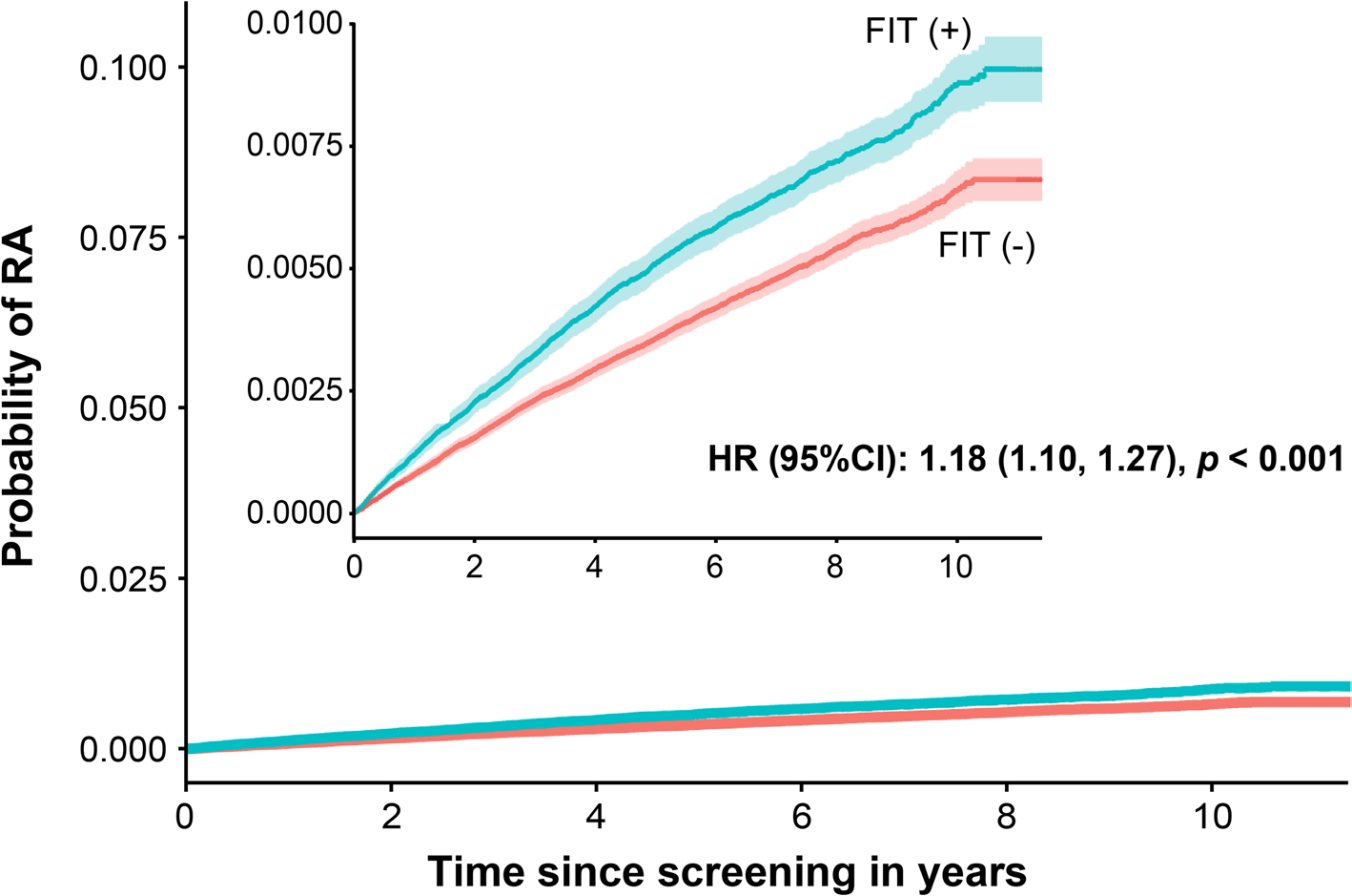
Kaplan-Meier analysis of the incidence of rheumatoid arthritis in the matched population. Insets present the identical data in an enlarged *y*-axis. *FIT* faecal immunochemical test, *HR* hazard ratio, *CI* confidence interval, *RA* rheumatoid arthritis

Since there were substantial differences in baseline characteristics between the FIT (+) and FIT (−) group, PSM was performed to reduce the difference between the groups. As shown in Table 7, after matching, the differences in clinical parameters disappeared. Kaplan–Meier analysis in the matched population revealed that the FIT (+) group still had a significantly higher risk of developing RA than the FIT (−) group (HR 1.18, 95% CI 1.10, 1.27, *p*<0.001, by the log-rank test) (Fig. 2).

**Table 7:** Baseline characteristics after propensity-score matching

	Total cohort ***n***=437,250	FIT (−) ***n***=291,500^a^	FIT (+) ***n***=145,750^a^	***p*** value	SMD
**Clinical parameter**					
Sex				0.227	0.0033
Male	246,455 (56.4)	164,490 (56.4)	81,965 (56.2)		
Female	190,795 (43.6)	127,010 (43.6)	63,785 (43.8)		
Age group at screening, years				0.738	<0.001
50–54	131,325 (30.0)	87,635 (30.1)	43,690 (30.0)		
55–59	92,338 (21.1)	61,530 (21.1)	30,808 (21.1)		
60–64	92,705 (21.2)	61,699 (21.2)	31,006 (21.3)		
65–69	57,884 (13.2)	38,527 (13.2)	19,357 (13.3)		
≥70	62,998 (14.4)	42,109 (14.4)	20,889 (14.3)		
BMI, kg/m^2^	24.1 [22.2, 26.1]	24.1 [22.1, 26.1]	24.1 [22.3, 26.1]	0.896	0.003
Smoking status				0.744	0.001
No	367,195 (84.0)	244,834 (84.0)	122,361 (84.0)		
Yes	70,055 (16.0)	46,666 (16.0)	23,389 (16.0)		
Alcohol drinking				0.541	0.002
No	340,638 (77.9)	227,013 (77.9)	113,625 (78.0)		
Yes	96,612 (22.1)	64,487 (22.1)	32,125 (22.0)		
Exercise				0.790	<0.001
No	134,288 (30.7)	89,487 (30.7)	44,801 (30.7)		
Yes	302,962 (69.3)	202,013 (69.3)	100,949 (69.3)		
Insurance type				0.223	<0.001
Medical aid	2,336 (0.5)	1,585 (0.5)	751 (0.5)		
NHI	434,914 (99.5)	289,915 (99.5)	144,999 (99.5)		
Comorbidities					
Hypertension				0.657	0.001
No	390,814 (89.4)	260,500 (89.4)	130,314 (89.4)		
Yes	46,436 (10.6)	31,000 (10.6)	15,436 (10.6)		
Diabetes				0.709	0.001
No	410,481 (93.9)	273,626 (93.9)	136,855 (93.9)		
Yes	26,769 (6.1)	17,874 (6.1)	8,895 (6.1)		
Dyslipidaemia				0.402	0.003
No	310,009 (70.9)	206,554 (70.9)	103,455 (71.0)		
Yes	127,241 (29.1)	84,946 (29.1)	42,295 (29.0)		
**Laboratory measures**					
Blood haemoglobin, g/dL	13.8 [12.8,14.9]	13.8 [12.8,14.9]	13.8 [12.8,14.9]	0.002	0.001
Serum creatinine, mg/dL	0.9 [0.8,1.0]	0.9 [0.8,1.0]	0.9 [0.8,1.0]	<0.001	0.005
Serum total cholesterol, mg/dL	198 [174,224]	198 [174,224]	198 [174,224]	0.304	0.004
Serum triglycerides, mg/dL	116 [81,167]	116 [82,166]	16 [81,167]	0.318	<0.001
Serum HDL-cholesterol, mg/dL	52 [44,61]	52 [44,61]	52 [44,62]	<0.001	<0.001
Serum LDL-cholesterol, mg/dL	118 [95,142]	118 [95,142]	18 [95,142]	0.058	0.003

Data presented are shown as median [interquartile range] or number (%)

*FIT* faecal immunochemical test, *SMD* standardised mean difference, *BMI* body mass index, *NHI* national health insurance, *HDL* high-density lipoprotein, *LDL* low-density lipoprotein

^a^A 1:2 matching of FIT (−) group was done with FIT (+) group

## Discussion

FIT is a non-invasive test used to detect blood in faeces and screen for CRC [29]. Nonetheless, Libby et al. [30] and Chen et al. [13] have indicated that occult blood in faeces could predict an increase in mortality that is not accounted for CRC among national health-examination recipients, probably representing a generalised inflammatory status. This is also highlighted by a number of studies that have demonstrated an association between positive FIT and the risk of developing chronic disorders, raising a possibility that FIT abnormality, without gross abnormal mucosal lesions, could be an indicator of subjects that are at risk of chronic diseases mirroring heightened inflammation [9]. In the present study, we classified participants undergoing FIT in a national CRC screening programme into FIT (+) and FIT (−) groups and compared the incidence of IMIDs. We selected participants that had undergone colonoscopic examination as a sequential test in the FIT (+) group, to rule out other causes that could lead to abnormal FIT. First, those with haemorrhoids, which are non-inflammatory mucosal lesions contributing to positive FIT [31], IBD, and CRC at baseline were excluded in the FIT (+) group. By further excluding those who developed CRC during the first year after screening, and those diagnosed with IBD within 6 months in both groups, we tried to exclude well-known causes that could result in positive FIT. Intriguingly, in the investigated IMIDs, we found that the incidence of RA was increased in participants with FIT (+) compared to those in the FIT (−) group, when time was taken into account. The IR before/after adjusting for sex and age between the two groups showed overlapping CIs and no significant difference. Nonetheless, we found that the FIT (+) group had a higher hazard, compared to the FIT (−) group in the Cox proportional hazards regression model because this model handles unequal follow-up times and the effects of other covariates. The replication of these results, even after PSM affirmed that FIT positivity is relevant to the development of RA.

The profound effect of the gut on the immune system has been well characterised. In particular, there is ample evidence illustrating that the risk of RA is heightened in the presence of gut mucosal abnormalities per se. Indeed, a previous study has shown that loss of intestinal mucosal integrity led to inflammation and dysbiosis, and recovery in the intestinal barrier function inhibited arthritis [32]. It was also found that alterations in the gut microbiota are responsible for gut mucosal inflammation and the development of murine arthritis [33]. In addition, there is evidence that inflammatory arthritis promotes injury of the gut barrier, suggesting that gut abnormalities have a pathogenic effect in the development of arthritis and vice versa [34]. Based on this, while it has been widely hypothesised that abnormal gut mucosa may be associated with the evolution of RA, this association has not been determined in the general population. To the best of our knowledge, this is the first study demonstrating elevated risk of RA with abnormal FIT results.

Annual faecal occult blood tests with FIT are recommended as a CRC screening test in South Korea [29, 35]. When FIT result is positive but the colonoscopy result is negative, there is no need for further testing unless there is anaemia or gastrointestinal symptoms [36]. Compared to the guaiac method, FIT is a test that allows sensitive detection of colonic and rectal bleeding [37]. Of note, an important strength of our study is that we excluded cases of cancer, IBD, and haemorrhoids that could cause bleeding, from the analysis through the KNCSP protocol using the colonoscopy results of the FIT positive group. The FIT (+) group included cases in which faecal haemoglobin was confirmed without macroscopic abnormalities of cancer, IBD, and haemorrhoids at colonoscopy. It is difficult to clearly explain the FIT (+) but colonoscopy negative group. We inferred that this finding may indicate systemic inflammation, as previously described [9]. Therefore, this study started with the assumption that the FIT (+) group would differ from the FIT (−) group, as they could potentially represent subclinical inflammation. Nonetheless, because there are also publications showing a relatively low positive predictive value of FIT positivity in both symptomatic and asymptomatic individuals, multiple factors that could affect in abnormal FIT should be also taken into consideration [38].

Our result underscores that in those with a positive FIT, articular symptoms should be vigilantly monitored and should be advised to consult a rheumatology specialist, since they are at greater risk of developing RA than those in the FIT (−) group. Additionally, the higher incidence of RA within the first year of abnormal FIT indicates that early referral to a rheumatology department is imperative when there is persistent, multiple joint pain, or those showing signs suggestive of RA. This seems particularly important because high clinical suspicion is a prerequisite for diagnosis of seronegative RA, which accounted for approximately 60% of cases in our study population. However, in contrast to RA, the relationship with SLE and PsA was not revealed herein. Although this may be due to the small number of participants developing SLE and PsA during the follow-up, the possibility also exists that the inclusion of a study population exclusively over the age of 50 could have also affected the study results.

We observed that 0.73% of participants were diagnosed with RA during the mean follow-up of 7.6 years. Of note, epidemiologic studies performed in the US and Sweden indicated that the incidence of RA was approximately 40/100,000 annually [39, 40], while the annual incidence of RA in South Korea was reported to be between 16.5 and 42.0/100,000 through a nationwide database [21]. Our results showed that the IR of RA was 9.56/10,000 PY, which was more than twice the rate seen in existing studies. In addition, the proportion of patients with seronegative RA, which usually accounts for 30–40% of RA cases, was higher than that of seropositive RA, showing a different pattern of disease subtypes than in the general population [41]. Even though the reason for this observation is unclear, this finding could be related to the lower rate of antibody detection in early RA; of interest, data from the ESPOIR cohort suggested that antibody detection rates in early RA could be as low as 50%, and that seronegative RA may be even more common than seropositive RA in early RA [42]. Additionally, the discrepancies in RA incidence, compared to the general population, appears to be mainly attributable to the study design. Generally, the incidence of RA is higher in the older, compared to the younger population; since participants in the national CRC screening programme were aged 50 at a minimum, this would have resulted in a substantially higher RA annual IR.

Evaluation of predictive factors of RA showed that positive FIT, female sex, age of under 70, and serum HDL-cholesterol < 60 mg/dL were associated with increased risk of RA occurrence, while alcohol had a negative correlation. This observation appears to be convincing given that RA is more common in women than in men and the incidence of RA peaks at the age of 50–60 and decreases after the age of 70 [43]. Moreover, HDL-cholesterol, which traditionally plays a protective role in the cardiovascular system, is also being increasingly understood to have an immunoregulatory effect by inhibiting the inflammatory response [44]. Therefore, the decrease in HDL-cholesterol could be relevant to augmented inflammation and lead to the development of RA. Alternatively, because it has been described in RA that reduced HDL-cholesterol levels are linked to increased inflammation, abnormal HDL-cholesterol levels could indicate a dysregulated status of immunity prior to the development of clinically evident synovitis [45]. Finally, studies have suggested that alcohol ingestion could negatively impact the incidence of RA and disease severity, which has also been observed in our results [46, 47].

There are several issues that should be considered as limitations of this study. First, although a large number of patients were included in this study, baseline characteristics were only considered in respect of the occurrence of IMIDs. Second, as all participants were 50 years or older, the impact of FIT on the incidence of IMIDs in younger individuals could not be assessed. Third, because multiple factors including medication (i.e. non-steroidal anti-inflammatory drugs, anticoagulants, antiplatelet therapy, glucocorticoids) can affect the FIT results, the possibility of other causes of abnormal FIT may not have been totally excluded. Fourth, there were missing data regarding occupation, lifestyle measures, comorbidities, and laboratory measures. Fifth, we did not know the colonoscopy results in the FIT (-) group because additional evaluation is not covered by the KNCSP. Sixth, the pre-FIT tests of participants could not be identified in this study, due to data unavailability. Seventh, polyps were included in the abnormal FIT group in our study. A large polyp may be a cause of FIT positivity, which could not be analysed. However, the pathological information of individual participants was protected from disclosure by the NHIS because of privacy issues; hence, it could not be used for analysis. Eighth, although we extracted subjects developing CRC, IBD, or IMIDs using the ICD-10 codes and either the medications of DMARDs or the registration in the rare intractable disease through validated definitions, there is a possibility that some of these patients might have alternative diagnoses. Ninth, our result should be interpreted with caution, not solely by relying on *p* values, since this analysis was conducted with substantially large samples; therefore, *p* values can be very low, regardless of statistical significance. Finally, validation using a different study sample will verify this study and give more profound results. Therefore, additional studies are warranted in the future to verify our results and provide better evidence that an abnormal FIT is linked to the occurrence of RA.

## Conclusions

In conclusion, using a large-scale nationwide cohort, we demonstrated that an abnormal FIT was associated with an increased incidence of IMID, particularly RA. The increased risk of RA in this population emphasises that regular screening and early referral to a specialist are necessary upon medical suspicion. Furthermore, this real-world evidence corroborates the understanding that FIT positivity contributes to the development of RA, which has a complex pathogenic mechanism, and may have possible future applications in the detection of early chronic diseases.

## Data Availability

Data cannot be shared publicly because of the sensitive nature of the data collected for this study in accordance with the Personal Information Protection Act of Republic of Korea. Data are available from the Korea National Health Insurance Sharing Service (contact via https://nhiss.nhis.or.kr, contact: +82-33-736-2432, 2433) for researchers who meet the criteria for access to confidential data.

## Abbreviations

BMI: Body mass index
CI: Confidence interval
CRC: Colorectal cancer
DMARD: Disease-modifying anti-rheumatic drugs
FIT: Faecal immunochemical test
HDL: High-density lipoprotein
HR: Hazard ratio
IBD: Inflammatory bowel disease
ICD-10: International Statistical Classification of Diseases, Tenth revision
IMIDs: Immune-mediated inflammatory diseases
IR: Incidence rate
KNCSP: Korean National Cancer Screening Program
LDL: Low-density lipoprotein
NHIS-NHID: National Health Insurance Sharing Service-National Health Information Database
PsA: Psoriatic arthritis
PSM: Propensity-score matching
PY: Person-years
RA: Rheumatoid arthritis
SD: Standard deviation
SLE: Systemic lupus erythematosus
